# Artificial intelligence in vaccine development: applications, implementation, and future directions

**DOI:** 10.3389/fcimb.2026.1870890

**Published:** 2026-07-15

**Authors:** Sastha N. Kumar, Raj Gondane, Shubham Mahindrakar, Sukrut Vyawahare, Renju Krishna, Sabarinath Subramaniam, Bipin G. Nair, Geetha B. Kumar, Aravind Madhavan, Nidheesh M, Pradeesh Babu

**Affiliations:** 1 Amrita School of Biotechnology, Amrita Vishwa Vidyapeetham, Kollam, India; 2 Peter MacCallum Cancer Centre, Melbourne, VIC, Australia; 3 Sivasakthi Science Foundation, Trivandrum, India

**Keywords:** antigen discovery, artificial intelligence (AI), epitope prediction, immunogen design, immunoinformatic, machine learning (ML), reverse vaccinology, structural vaccinology

## Abstract

Vaccination stands as one of the most transformative interventions in the history of human civilization. In medicine, vaccination stands as a cornerstone that has saved countless lives across generations. Nevertheless, conventional vaccine development remains encumbered by prolonged timelines, substantial financial investment, and high attrition rates particularly during late-stage clinical trials underscoring the urgent need for more efficient and systematic approaches. In recent years, artificial intelligence (AI) has emerged as a transformative force across the biomedical sciences, offering unprecedented computational capacity to process and interpret complex biological datasets. The convergence of AI with vaccinology represents a significant methodological advancement which has the potential to fundamentally redefine the vaccine development paradigm. AI integrates advances in machine learning, multi-omics data analysis, and high-performance computing to accelerate antigen discovery, epitope prediction, immunogen design, and clinical evaluation. This development represents a paradigm shift toward faster, more precise, and scalable strategies for vaccine development. This review critically examines the current landscape of AI applications in vaccine development, with particular emphasis on recent advancements, translational challenges, and the prospective role of AI in shaping the future of immunization science.

## Methods: PRISMA based data curation for artificial intelligence in vaccine development:

### Search strategy

This semi-systematic narrative review was conducted in accordance with the Preferred Reporting Items for Systematic Reviews and Meta-Analyses (PRISMA) guidelines, adapted for narrative and scoping reviews. A comprehensive electronic literature search was performed across four major bibliographic databases: PubMed/MEDLINE, Scopus, Web of Science, and Google Scholar. The search was conducted for articles published between January 2015 and December 2025 to capture the most relevant advances in the application of artificial intelligence (AI) to vaccine development.

### Search terms

The following key search terms were used, individually and in combination using Boolean operators (AND, OR): “Artificial Intelligence”, “Machine Learning”, “Deep Learning”, “Vaccine Development”, “Vaccinology”, “Epitope Prediction”, “Antigen Discovery”, “Reverse Vaccinology”, “Structural Vaccinology”, “Immunoinformatics”, “mRNA Vaccines”, “Clinical Trial Optimization”, “Protein Structure Prediction”, “AlphaFold”, “NetMHCpan”, “Multi-omics Integration”, “Vaccine Manufacturing”, and “Pharmacovigilance”. Searches were limited to peer-reviewed original research articles, reviews, and systematic reviews published in English.

### Inclusion and exclusion criteria

Studies were included if they: (i) described the development, validation, or application of AI/ML tools in any stage of vaccine development; (ii) were published in English between 2015 and 2025; (iii) provided sufficient methodological detail to evaluate AI performance or applicability; and (iv) were peer-reviewed primary research articles, review articles, or methodological papers. Studies were excluded if they: (i) were not focused on AI or computational methods; (ii) were published before 2015 or in languages other than English; (iii) were conference abstracts, editorials, or opinion pieces without substantive methodological content; or (iv) provided insufficient data for meaningful synthesis.

### Study selection and data extraction

**Figure 1:**
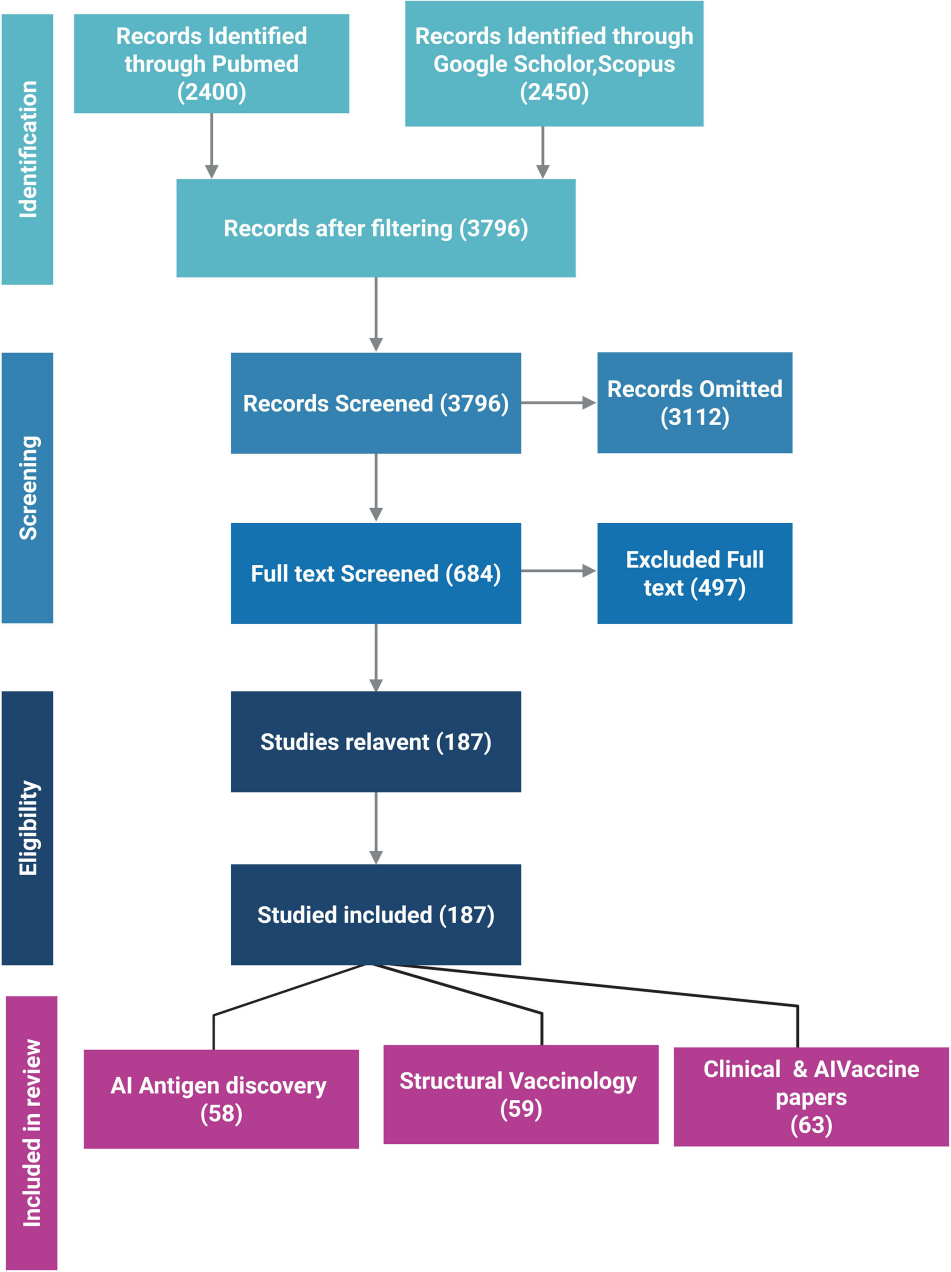
PRISMA-style flow diagram illustrating the literature search and study selection process for this semi-systematic narrative review on AI applications in vaccine development. Created in BioRender. Nair, B. (2026) https://BioRender.com/9icyzat.

Following de-duplication, titles and abstracts of all identified records were screened independently by two reviewers to assess eligibility. Full-text articles of potentially relevant studies were subsequently retrieved and assessed against the pre-defined inclusion and exclusion criteria. Disagreements at both stages were resolved through discussion and consensus. Data were extracted from included studies into a structured template, capturing information on AI model type, application domain within vaccine development, validation approach, performance metrics, and key findings. The entire selection process is summarized in the PRISMA-style flow diagram below (**Figure 1**).

## 1 Introduction

Vaccination remains one of the most effective public health interventions ever developed. The creation and global distribution of these vaccines represent a cornerstone of modern medicine, credited with preventing millions of deaths annually and successfully controlling numerous infectious diseases (Rappuoli et al., 2014). The unprecedented speed at which emerging pathogens such as SARS-CoV-2 have spread across populations has exposed fundamental limitations in conventional vaccine development paradigms. The established pathway, while rigorous and safety oriented, is notoriously slow, costly, and fraught with high attrition rates, frequently taking a decade or longer to transition a candidate from the laboratory to clinical settings (Kaushik et al., 2023).This inherent inefficiency poses a significant challenge during global health crises where rapid response is paramount (Vitorino, 2024). 

### 1.1 The evolution of antigen discovery: from empirical observation to in silico design

**Figure 2:**
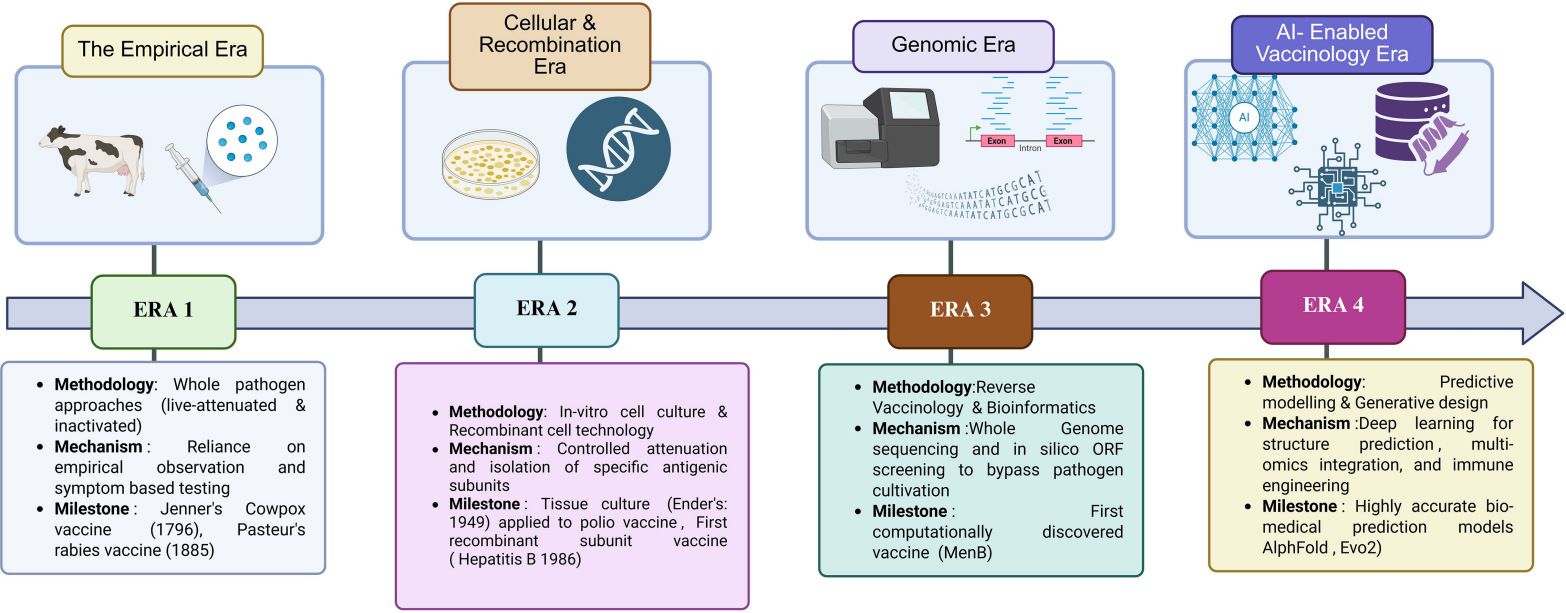
Timeline of key milestones in antigen discovery and vaccine development, illustrating the transition from empirical methods to AI-driven design Today, vaccinology is undergoing a fourth transition: moving from static genomic screening to dynamic, predictive modeling. Recent advances in artificial intelligence (AI) and machine learning (ML) are catalyzing a profound shift in vaccinology. AI-driven approaches are reshaping vaccine development into a predictive, data-integrated, and systems-level discipline (Elfatimi et al., 2025). This transformation is achieved by leveraging high-throughput data processing, predictive modeling, and pattern recognition across vast biological datasets, including genomic, proteomic, and clinical information. AI offers a powerful toolkit to accelerate timelines, reduce costs, and increase the probability of success in vaccine design and development (Esmaeilpour et al., 2026). Created in BioRender. Nair, B. (2026) https://BioRender.com/9xrnzgd.

To understand the necessity of integrating advanced computational tools into this pipeline, it is important to trace how the timeline of vaccine development has been historically characterized by distinct methodological eras (**Figure 2**). The first generation of vaccines relied entirely on empirical observation and the use of whole organisms. Beginning with Edward Jenner’s use of cowpox in 1796 and advancing through Louis Pasteur’s live-attenuated vaccines in the late 19th century, this era established the foundational concept of immunological memory (Riedel, 2005). However, utilizing the entire antigenic collection of a pathogen often resulted in unpredictable safety profiles and high reactogenicity. A major transition occurred in the mid-20th century with the advent of mammalian cell culture. The ability to propagate viruses *in vitro* allowed for the controlled attenuation of pathogens, directly facilitating the development of staple viral vaccines for diseases like polio, measles, mumps, and rubella (Kumru et al., 2014). Despite this progress, antigen discovery remained fundamentally constrained by the need to physically cultivate the pathogen a significant bottleneck for highly mutable or fastidious organisms.

By the 1980s, the integration of molecular biology and recombinant DNA technology shifted the paradigm once again. Instead of relying on whole pathogens, scientists isolated specific genetic sequences encoding protective antigens, exemplified by the recombinant Hepatitis B vaccine (Zhao et al., 2020). This transition to purified subunit vaccines significantly improved safety profiles but still required exhaustive biochemical screening. The late 1990s introduced a third major milestone with the birth of “reverse vaccinology”. Driven by rapid advancements in whole-genome sequencing, researchers bypassed pathogen cultivation entirely, instead mining genomes *in silico* to identify open reading frames (ORFs) encoding surface-exposed proteins. This approach famously led to the Bexsero vaccine against *Neisseria meningitidis* serogroup B (MenB) a pathogen that had previously evaded traditional vaccine efforts (Vernikos and Medini, 2014).

In this Review, we examine how AI is reshaping vaccine development across all stages, from antigen discovery and immunogen engineering to clinical translation, manufacturing, and regulatory science (Kaushik et al., 2023). We highlight how AI-driven systems transform decision-making, reduce uncertainty, and open pathways toward precision and equitable vaccination strategies (Faiyazuddin et al., 2025).

## 2 Conventional vaccine development pipelines: structure, challenges, and limitations

Fundamentally, vaccines operate by introducing a harmless component of a pathogen such as an antigen or genetic material into the body. This exposure trains the host’s immune system to recognize the threat and mount a rapid, targeted defence, thereby establishing long-term immunological memory against future infections (Montero et al., 2024).To achieve this, Traditional vaccine development follows a largely linear trajectory meticulously designed to ensure the highest standards of safety and efficacy. Historically, rather than focusing on generalized clinical trial phases, traditional vaccinology is anchored in extensive, specialized laboratory development.

**Figure 3:**
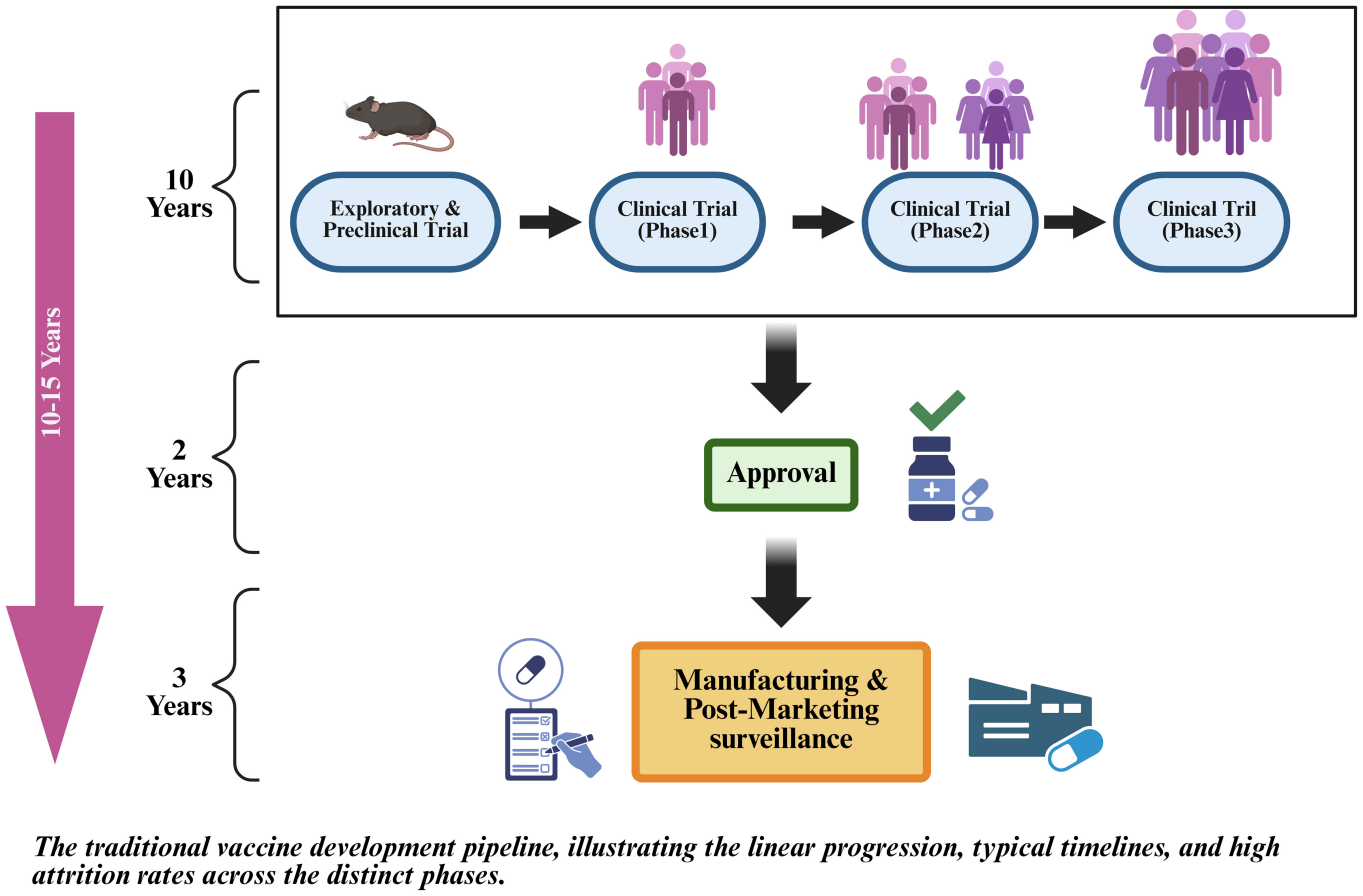
The traditional vaccine development pipeline. Figure explains traditional vaccine development pipeline showing the sequential stages involved in vaccine discovery, clinical evaluation, regulatory approval, and post-marketing surveillance. The figure illustrates the typical timeline associated with exploratory and preclinical studies, Phase I–III clinical trials, regulatory approval, and manufacturing/post-marketing surveillance, highlighting the lengthy and linear nature of conventional vaccine development. Created in BioRender. Nair, B. (2026) https://BioRender.com/h9a1z0b.

The core of this conventional pipeline begins with the empirical discovery and isolation of a specific immunogenic target, or antigen. This discovery process as mentioned in (**Figure 3**) traditionally requires the physical isolation and *in vitro* cultivation of the infectious agent, followed by biochemical lysis and fractionation (Pawar et al., 2026). Researchers systematically screen these crude protein fractions against convalescent human or animal sera using immunoassays (such as Western blotting or ELISAs) to identify which specific surface proteins or secreted toxoids elicit a strong antibody-binding response. Once a candidate antigen is biochemically identified, the development phase transitions to recombinant engineering. This involves isolating the specific genetic sequence encoding the antigen, cloning it into a plasmid vector, and introducing it into an expression system (e.g., *Escherichia coli*, yeast, or mammalian cell lines). The target antigen is then expressed at scale and subjected to rigorous downstream purification, utilizing techniques like affinity or size-exclusion chromatography (Sanyal, 2022). Finally, the purified antigen enters the formulation stage, requiring the empirical optimization of specific adjuvants and stabilizers to ensure both a robust immune response and shelf-life stability. The candidate advances to pre-clinical animal models only after successfully completing these exhaustive, laboratory-intensive milestones, a linear progression that frequently requires years of empirical benchwork (Shi et al., 2025).

While this traditional pipeline prioritizes safety and rigor, it is characterized by prolonged timelines, escalating costs, and high late-stage failure rates (Pawar et al., 2026). Attrition is particularly pronounced during clinical development, where unanticipated immunogenicity failures, safety concerns, or insufficient efficacy frequently emerge only after substantial investment (Roberts et al., 2014). Another major limitation is its reliance on empirical iteration. The exploratory and discovery phase focuses on identifying immunogens capable of eliciting protective immunity, including attenuated or inactivated pathogens, protein subunits, or nucleic acid–based constructs (Brisse et al., 2020). This stage is challenged by the immense biological complexity of host–pathogen interactions and the vast antigenic search space, resulting in high attrition rates and heavy reliance on empirical, trial-and-error screening (Zhou et al., 2025). Promising candidates then advance to pre-clinical testing, where safety, immunogenicity, and dosing parameters are evaluated through *in vitro* assays and animal models. Although essential for risk mitigation, these preliminary stages are time-consuming and provide only limited predictive insight into human immune responses (Green and Al-Humadi, 2016). Antigen selection, dose optimization, and trial design often depend on heuristic reasoning and incremental experimentation, resulting in narrow exploration of the immunological design space. Moreover, conventional pipelines struggle to adapt dynamically as new biological insights, pathogen variants, or population-level data emerge.

Clinical development constitutes the most resource intensive phase, typically spanning multiple years and progressing through phased human trials to assess safety, immunogenicity, dosing, and ultimately efficacy at population scale. Successful candidates proceed to regulatory review, during which comprehensive evidence of safety, efficacy, and manufacturing quality is evaluated before licensure (Singh and Mehta, 2016). Following approval, vaccines enter large-scale manufacturing and post-market surveillance, where real-world effectiveness and rare or long-term adverse events are monitored continuously. Collectively, this linear and compartmentalized framework, while ensuring rigorous safety standards, remains poorly suited to rapid outbreak response and highlights the need for more predictive, adaptive, and integrated development strategies (Thakkar et al., 2023).

Thus, when we critically analyse the traditional vaccine development, we can say that the traditional pipelines have relied mostly on experimental approaches, which are not only costly but also highly time-consuming. Even preliminary trials that are only remotely feasible demand significant investment in specialized equipment, infrastructure, and resources. This dependence on conventional experimentation slows progress and creates barriers to rapid innovation, highlighting the urgent need for more efficient, computationally driven strategies to complement and accelerate the development process.

## 3 AI-driven transformation of vaccine development pipelines

Artificial Intelligence has come a long way from the initials days and is applied in everyday life scenarios; in vaccine development AI is playing an increasingly central role in vaccine development and is expected to expand further. The application of AI in vaccine development systems has potential to vastly increase transform the traditional, linear trajectory of vaccine development into an iterative, closed-loop learning framework driven by continuous data integration. Within this framework, computational predictions guide experimental design, while the resulting empirical data continuously refine the underlying models, allowing insights to propagate across the pipeline in near real time (Anderson et al., 2025). Rather than treating discovery, development, and deployment of vaccines as isolated phases AI systems enables continuous feedback from initial stages of molecular interactions to population-level outcomes after production (Zhao and Wang, 2025). This continuous feedback loop system ensures that at each iteration, the pipeline for vaccine development is updated based on its output, further enhancing the development process.

The transformation from traditional to AI-integrated vaccine pipeline is due to three major developments, the first is the exponential growth in biological and clinical data, the second is the advances in machine learning architectures capable of modelling complex, nonlinear systems, and scalable computational infrastructure that supports real-time analysis (Maleki Varnosfaderani and Forouzanfar, 2024). By replacing slow, empirical methods with rapid, data-driven computational approaches AI promises to significantly reduce timelines and failure rates. This represents a fundamental shift from a linear, trial-and-error process to an accelerated, predictive, and targeted methodology. Together, these elements allow vaccinology to transition from trial-and-error workflows toward predictive, systems-level design (Chopra et al., 2023).

The AI-based vaccine developmental approaches begin from the initial discovery phase of vaccine candidate. A primary bottleneck in conventional vaccinology is the identification of suitable antigens. AI-powered approaches, particularly those in the fields of immuno-informatics and reverse vaccinology, can analyse the genomic and proteomic data of a pathogen to perform large-scale *in silico* screening for proteins and epitopes that are likely to serve as effective immunogens (Parihar et al., 2022). Once potential antigens are identified, AI models assist in structural optimization and formulation design to ensure stability and targeted delivery. Following this design stage, the predictive power of AI extends into the pre-clinical and clinical phases. Machine learning algorithms can model the potential immunogenicity and safety of a vaccine candidate, allowing researchers to prioritize the most promising designs (Joshi and Sheth, 2025). During human trials, AI tools can optimize cohort selection, stratify patient populations for more robust data, and continuously analyze emerging evidence to inform trial modifications in real-time (Chopra et al., 2023). By facilitating the design of smaller, shorter, and more efficient clinical trials, AI improves statistical power and accelerates the overall timeline for regulatory submission and approval. This streamlined process directly addresses the critical need for rapid response during public health emergencies (Podichetty et al., 2025).

When analysing the development of such pipelines it’s important to understand the fact that that these systems function as tools designed to uncover patterns that may not be immediately apparent to human researchers. On one hand, the identification of hidden correlations can accelerate the discovery of novel drug candidates and vaccine formulations. On the other hand, if these patterns are not grounded in accurate biological understanding, they risk leading to misleading conclusions or ineffective therapeutic strategies. Thus, while AI enhances the scope of exploration, its outputs must be carefully validated through rigorous biological and clinical frameworks to ensure meaningful and safe advancements in vaccine development.

The effective integration of artificial intelligence into vaccine development is underpinned by robust computational infrastructure capable of supporting large-scale data processing and complex model training. High-performance computing resources, including graphics processing units and high-memory architectures, enable rapid analysis of genomic, proteomic, and structural datasets that would otherwise be computationally prohibitive. In parallel, cloud-based platforms provide scalable and flexible environments for distributed computation, facilitating rapid model iteration and international collaboration. One such example proved to be critical is during the COVID-19 pandemic, allowing vaccine candidates to be designed, optimized, and evaluated at unprecedented speed. Emerging hardware developments, including specialized accelerators and exploratory quantum systems, further expand the computational horizon for simulating molecular interactions and immune dynamics (Anderson et al., 2025). Equally important are integrated software ecosystems that connect diverse stages of the vaccine design workflow, from structural modeling to immunogenicity prediction and molecular optimization (Olawade et al., 2024). Advances in protein structure prediction have enabled structure-guided vaccine design to proceed without prolonged experimental bottlenecks, while computational immunology platforms support systematic identification of immune-relevant antigenic features.

AI models trained on biological data are opening new possibilities for vaccine development. When combined with computer-based molecular modeling and optimization tools, they allow scientists to test many ideas before moving to lab experiments (Imani et al., 2024). With powerful computing systems and software that can work together across borders, vaccine research is becoming faster, more predictive, and more collaborative worldwide. This means AI-driven vaccinology can work effectively at both the molecular level and across large populations, though success will still depend on ensuring data quality, transparency, and fair access to these technologies.

## 4 AI-enabled vaccine discovery and design

AI-enabled vaccine discovery and design represent a major shift from traditional trial-and-error approaches toward data-driven innovation. By leveraging machine learning models trained on genomic, proteomic, and immunological datasets, researchers can predict antigenic targets with higher accuracy and identify epitopes most likely to elicit protective immune responses. In silico modeling allows rapid simulation of molecular interactions between candidate antigens and host immune receptors, reducing reliance on costly and time-consuming laboratory screening. The major models available at the time of writing for biological uses include the following as summarised in (**Table 1**). 

**Table 1:** Bio-models and their role in vaccine development.

Model/framework	Primary usage	Advantages	Limitations	Examples of antigen discovery (antigen name & target disease)	Application to vaccine development
*AlphaFold 3*	Protein structure and biomolecular complex prediction.	High structural accuracy; supports conformational epitope and antibody–antigen analysis.	Requires wet-lab validation; limited for membrane dynamics and glycosylation.	RH5/Pfs48/45 (Malaria), Spike Protein (COVID-19)	Structural vaccinology, epitope prioritization, and immunogen design.
*RoseTTAFold All-Atom*	All-atom assembly prediction across proteins, nucleic acids, and small molecules.	Broad molecular scope; supports integrated structural reasoning.	Variable performance; requires downstream benchmarking.	Envelope Glycoprotein gp120 (HIV-1), Hemagglutinin with cofactors (Influenza)	Structural validation of candidate antigens and immunogen design.
*Chai-1*	Multimodal structure prediction across diverse biomolecular systems.	State-of-the-art benchmark performance; useful as a cross-validation tool.	Outputs remain hypothetical without experimental confirmation.	Spike-mEpitope complexes (SARS-CoV-2), EGFR/HER2 extracellular domains (Cancer Immunotherapy)	Antigen selection, epitope presentation analysis, and immunogen refinement.
*RFdiffusion*	Generative design of novel protein backbones, scaffolds, and binders.	Effective for *de novo* design and scaffold generation.	Designed proteins may fail folding, stability, or expression screens.	Prefusion F Protein (Site IV/V) Scaffolds (RSV), HA Stem Binders (Universal Influenza)	Epitope-focused vaccine design and antigen-display system engineering.
*ESM3*	Protein language modeling over sequence, structure, and function.	Strong sequence–structure–function reasoning; supports protein optimization.	Outputs require biochemical and immunological verification.	Optimized RBD/Spike variants (Pan-Coronavirus), Stabilized Env Trimers (HIV)	Antigen redesign, sequence optimization, and immunogen variant exploration.
*Evo 2*	Genomic foundation modeling across DNA, RNA, and proteins at scale.	Genome-scale conservation analysis and variant-aware biological reasoning.	Not immune-specific; requires integration with epitope and structure tools.	Conserved Polymerase/Surface Glycoproteins (Universal Influenza, Lassa Fever)	Reverse vaccinology, conservation analysis, and escape-resistant antigen selection.
*NetMHCpan 4.1/NetMHCIIpan 4.1*	Peptide–MHC class I and II binding prediction across HLA alleles.	Widely validated; directly applicable to T-cell epitope prioritization.	Binding affinity alone does not confirm *in vivo* processing or protection.	NY-ESO-1/MAGE-A3 Neoantigens (Melanoma), Gag/Pol Epitopes (HIV/AIDS)	T-cell epitope selection in subunit and multiepitope vaccine design.
*BepiPred 3.0*	Linear B-cell epitope prediction from protein sequence.	Rapid first-pass screening for antibody-targeted epitope regions.	Insufficient for conformational epitopes without structural integration.	Envelope (E) Protein linear domains (Zika/Dengue Virus), Major Outer Membrane Protein (MOMP) (Chlamydia)	Initial B-cell epitope filtering integrated with conservation analysis.

### 4.1 Antigen discovery and epitope prediction

**Figure 4:**
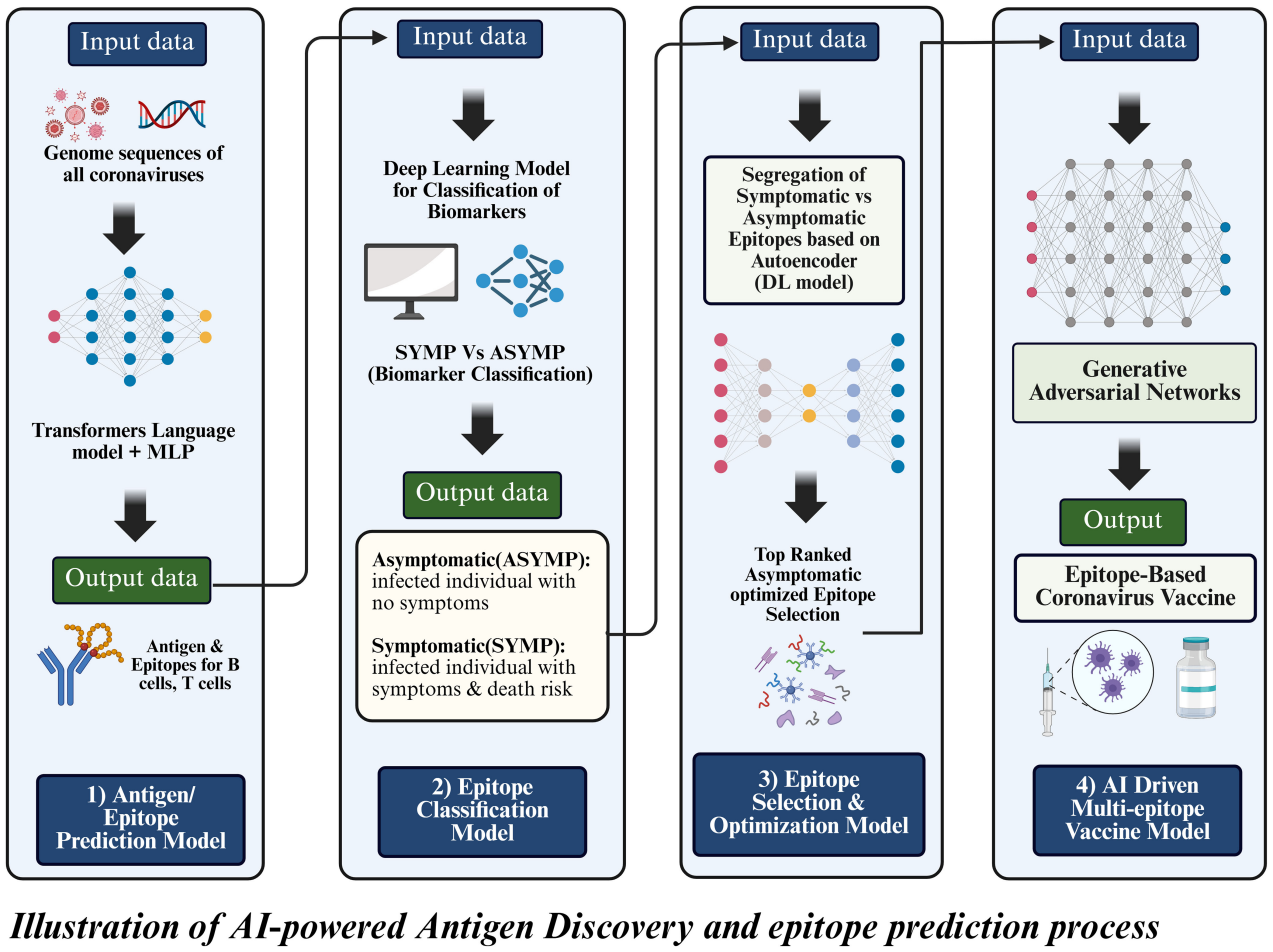
Illustration of AI-powered antigen discovery and epitope prediction process. The figure illustrates a sequential deep learning–based pipeline integrating antigen/epitope prediction, biomarker classification, epitope optimization, and AI-driven vaccine design. Initially, coronavirus genomic sequence data are analysed using transformer language models and multilayer perceptron (MLP) architectures to identify antigenic epitopes targeting B cells and T cells. The identified epitopes are subsequently classified into symptomatic and asymptomatic biomarker groups using deep learning algorithms, followed by autoencoder-based epitope optimization and generative adversarial network (GAN)-assisted multi-epitope vaccine design. Created in BioRender. Nair, B. (2026) https://BioRender.com/t9z9nb3.

The foundational step in rational vaccine design is the identification of suitable antigens and the specific epitopes within them that are recognized by the immune system. AI excels at this task by sifting through vast amounts of genomic and proteomic data to prioritize targets with the highest probability of inducing a protective immune response. A key application is the prediction of B-cell and T-cell epitopes. Machine learning models, trained on large datasets of experimentally verified epitopes, can identify the sequence of motifs and structural features associated with immunogenicity as illustrated in (**Figure 4**) (Bukhari et al., 2022). This has given rise to a suite of immunoinformatic tools that can score, and rank protein sequences based on their predicted antigenicity. Modern epitope prediction has evolved beyond simple motif matching toward integrative models that incorporate peptide processing, MHC binding, structural accessibility, and population-level HLA diversity. Deep learning frameworks now model the full antigen presentation pathway, improving predictions of CD8^+^ and CD4^+^ T-cell responses across diverse human populations. Similarly, structure-aware models enable identification of conformational B-cell epitopes that dominate neutralizing antibody responses but are poorly captured by linear sequence analyses.

For instance, platforms like VaxiJen, and more advanced ML-based tools like Vaxign-ML and NetMHCpan, provide researchers with a computational framework to narrow down thousands of potential proteins to a manageable list of high-priority candidates for experimental validation, thereby significantly accelerating the discovery phase (Chen et al., 2022). 

### 4.2 T-cell epitope prediction and antigen presentation modeling

T-cell epitope prediction is a cornerstone of rational vaccine design, as it enables the identification of peptide fragments capable of binding major histocompatibility complex (MHC) molecules and eliciting cellular immune responses. Modern computational models learn sequence motifs, biochemical properties, and structural constraints that govern peptide–MHC interactions (Basmenj et al., 2025; Shawan et al., 2023). Among the most widely used tools is NetMHCpan, which employs artificial neural networks trained on large-scale peptide–MHC binding affinity data and mass-spectrometry-derived eluted ligand datasets. This approach enables robust prediction of both CD8^+^ and CD4^+^ T-cell epitopes across a broad range of human leukocyte antigen (HLA) alleles (Grewal et al., 2024). Complementing this, MHCflurry (O’Donnell et al., 2020) uses deep learning architectures to predict MHC class I binding affinity while also incorporating antigen processing features such as proteasomal cleavage and peptide presentation likelihood.

Beyond binding affinity alone, integrated models aim to capture the full antigen processing pathway. NetCTLpan (Stranzl et al., 2010) combines machine-learning predictions of proteasomal cleavage, transporter associated with antigen processing (TAP) transport, and MHC binding to identify cytotoxic T-cell epitopes more accurately. More recently, deep neural network–based approaches such as DeepHLApan have leveraged sequence encoding and embedding strategies to model complex peptide–HLA interaction patterns. Together, these tools enable the systematic prioritization of T-cell epitopes that can stimulate effective cellular immunity across genetically diverse populations, supporting the development of broadly protective vaccines.

Cancer vaccines represent an important example of antigen-based immunotherapeutic vaccine development, as they aim to stimulate antitumor immunity through the delivery of tumor-associated or tumor-specific antigens. Recent progress in mRNA-based cancer vaccines, including clinical advances reported by Moderna in late-stage melanoma, demonstrates the translational potential of antigen-guided vaccine platforms beyond infectious disease (Zhang et al., 2026).

### 4.3 B-cell epitope prediction and antibody recognition mechanisms

B-cell epitope prediction focuses on identifying regions of antigens that are accessible and capable of binding antibodies, thereby driving humoral immune responses. These epitopes can be either linear, consisting of contiguous amino acid sequences, or conformational, formed by spatially proximate residues brought together through protein folding. Computational models integrate sequence features, surface accessibility, flexibility, and structural information to predict such antibody-binding regions. BepiPred is a widely used tool that combines hidden Markov models with machine-learning classifiers trained on experimentally validated B-cell epitope datasets, enabling improved prediction of linear antibody targets.

Early neural-network-based approaches such as ABCpred (Saha and Raghava, 2006) demonstrated the feasibility of learning epitope-associated sequence patterns using fixed-length peptide windows. However, as most potent neutralizing antibodies recognize discontinuous epitopes, structure-aware methods have become increasingly important. DiscoTope (Høie et al., 2024) addresses this challenge by leveraging three-dimensional protein structures and amino-acid propensity scores to predict conformational B-cell epitopes. These computational approaches, particularly when integrated with structural modelling tools, allow vaccine designers to identify and optimize antigen regions that are most likely to induce strong and durable antibody responses.

## 5 Immunogen engineering and structural vaccinology

Beyond identifying natural targets, AI is instrumental in the rational design and *de novo* creation of optimized vaccine immunogens. This is particularly valuable for modern platforms like subunit or mRNA vaccines, where the antigen sequence can be precisely engineered. Deep learning models, including Generative Adversarial Networks (GANs) and Variational Autoencoders (VAEs), can generate novel protein sequences that do not exist in nature but are optimized for enhanced stability, expression, or immunogenicity (Ding et al., 2022). Furthermore, the revolution in protein structure prediction, spearheaded by tools like AlphaFold, enables highly accurate structure-based vaccine design (Jumper et al., 2021). By providing precise 3D models of target antigens, these tools allow scientists to visualize critical epitopes and engineer the immunogen to ensure these sites are optimally presented to the immune system. This integration of generative and predictive AI streamlines the bioengineering of vaccine candidates that are purpose-built for safety and efficacy. Recent advances in lipid nanoparticle-based RNA delivery have also expanded the therapeutic landscape beyond conventional mRNA vaccines, supporting the broader relevance of RNA nanomedicine in cancer immunotherapy. For example, PSMD1-targeted small interfering RNA delivered through lipid nanoparticles showed potent antitumor activity in multiple myeloma models by reducing tumor growth, overcoming proteasome inhibitor resistance, and prolonging survival in preclinical xenograft systems (Du et al., 2026).

A critical challenge in vaccine development lies in the precise design of epitopes using existing technologies. Artificial intelligence offers powerful solutions to overcome this barrier by enabling more accurate epitope prediction and optimization. These AI-driven approaches can be further strengthened through immune simulations, modulation strategies, and diverse applications, ultimately accelerating the development of effective and targeted vaccines.

### 5.1 AI-enabled structural vaccinology and protein design

**Figure 5:**
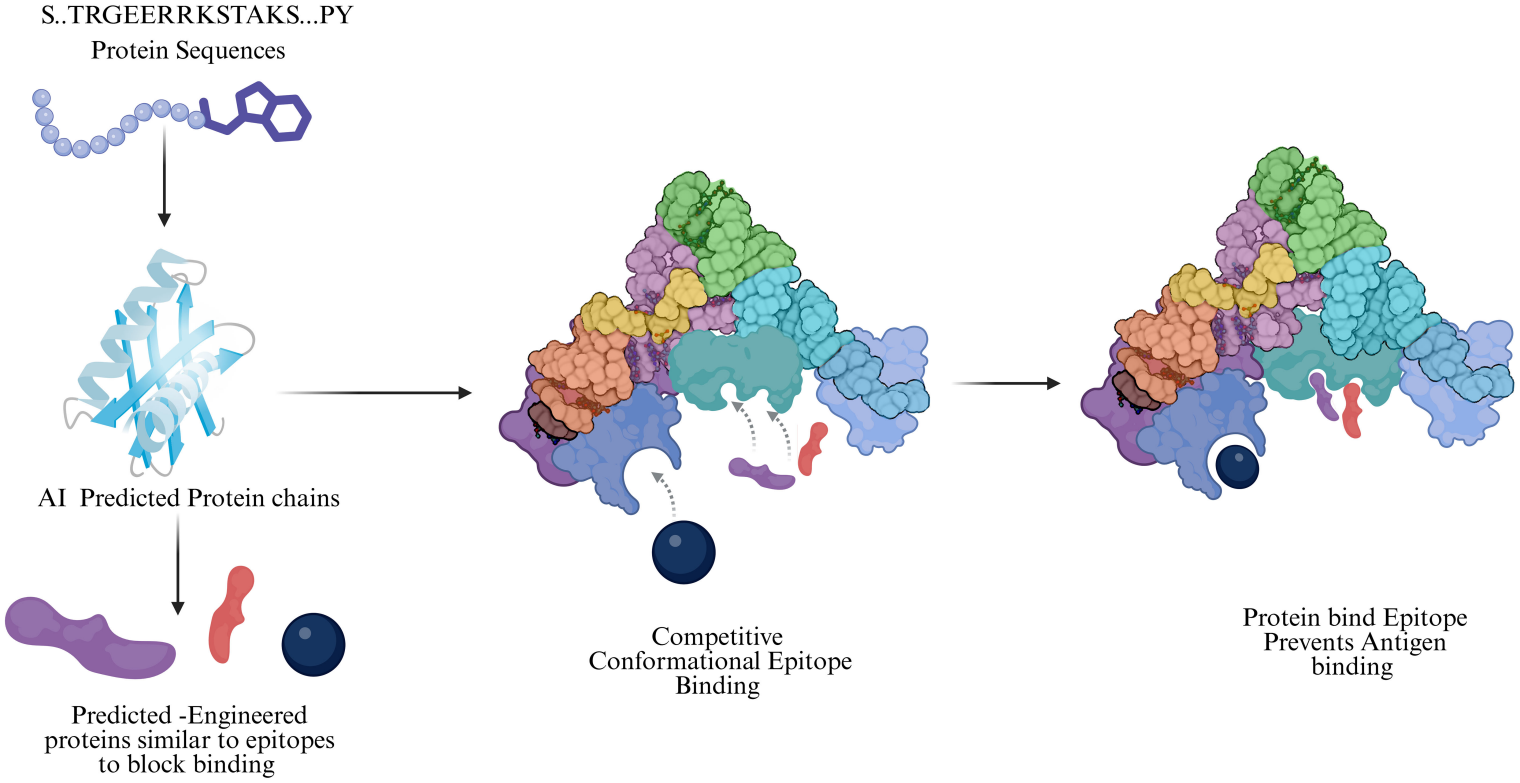
The AI- engineered development pipeline. The figure explains AI-engineered therapeutic development pipeline illustrating the use of artificial intelligence for protein structure prediction, epitope identification, and competitive binding-based therapeutic design. The workflow demonstrates how protein sequence data are processed using AI-based structural prediction tools to generate engineered proteins capable of targeting conformational epitopes and preventing antigen binding. The figure highlights the progression from computational sequence analysis to structure-guided inhibitor design and epitope-specific interaction. Created in BioRender. Nair, B. (2026) https://BioRender.com/lsadeg8.

Protein structure is the primary determinant for antigenicity, especially for neutralizing antibody that target conformational epitopes. High accurate structural prediction tools for protein help transform structural vaccinology from a niche discipline into a central pillar of vaccine design. The advent of high-accuracy AI-driven structure prediction models, most notably AlphaFold and RoseTTAFold, has enabled a new era of structural vaccinology (Jumper et al., 2021). Deep learning models have advanced to the point where they can predict protein structures directly from sequence data with near-experimental accuracy as illustrated in (**Figure 5**). Unlike traditional pattern-recognition approaches, modern biological models incorporate the biological context in which they are trained. This means they not only identify structural patterns but also provide insights into the underlying biological significance, offering explanations for why these structures exist. Such context-aware modeling enhances our ability to design vaccines by linking structural predictions to functional immunological outcomes. 

When choosing between AlphaFold2 (AF2) and RoseTTAFold for vaccine design, the performance gap between them is not trivial and has direct consequences for how reliably one can model the epitopes that matter most. AF2 encodes pairwise residue relationships through multiple rounds of attention-based refinement, which allows it to resolve long-range contacts across the protein with high fidelity. A median TM-score of 0.96 on the CASP14 benchmark reflects this (Bertoline et al., 2023). RoseTTAFold processes sequence, distance, and coordinate information simultaneously through three parallel tracks, a design that reduces memory requirements and speeds up inference, but at a meaningful cost to accuracy: its CASP14 TM-score in single-sequence mode drops to 0.68 (Hýsková et al., 2026). That 0.28 TM-score difference may appear modest in the abstract, but in practice it shows up most acutely in the regions that are hardest to model and most important for vaccines flexible surface loops, disordered linkers, and the exposed residues that form discontinuous conformational epitopes targeted by broadly neutralizing antibodies. When the goal is to stabilize a viral fusion protein in its prefusion conformation, or to transplant a conserved neutralizing site onto an engineered scaffold, small structural inaccuracies propagate into misrepresented epitope geometry and, ultimately, suboptimal antibody responses. ESMFold sits between the two tools in accuracy (TM-score 0.95) and has a practical advantage of its own: it runs on a single sequence without requiring a multiple-sequence alignment, which matters when working with novel or poorly characterized antigens where evolutionary data are limited. In many laboratories, these tools are now used in sequence rather than interchangeably. RoseTTAFold for an initial pass across a large panel of candidates, followed by AF2 or ESMFold for detailed structural analysis of the shortlisted antigens allowing teams to balance throughput against resolution depending on the stage of the design process (Bertoline et al., 2023). The quantitative performance differences across these tools are summarized in **Table 2**.

**Table 2:** Quantitative synthesis and comparative analysis of AI tools for vaccine development.

AI application domain	Model/tool	Primary function	Performance metric(s)	Dataset/scope	Aggregated/benchmark finding	Computational features	Vaccine application	Key limitation	Source
Protein Structure Prediction	AlphaFold2	Protein 3D structure prediction from sequence	TM-score 0.96; RMSD 1.30 Å	1,337 PDB structures deposited post-training cutoff	Highest structural accuracy among compared models	Slower; MSA-dependent	Structural vaccinology, antigen modelling, epitope mapping	Reduced accuracy for intrinsically disordered proteins (IDPs) and NMR structures	Hýsková et al. (2026
Protein Structure Prediction	ESMFold	Fast protein structure prediction without MSA	TM-score 0.95	1,337 PDB structures deposited post-training cutoff	Slightly lower accuracy than AlphaFold2 but substantially faster	10–30× faster than AF2; alignment-free	Large-scale antigen screening	Lower accuracy than AF2 for structured proteins	Hýsková et al. (2026)
Protein Structure Prediction	RoseTTAFold	Protein structure prediction from sequence	TM-score 0.68 (single-sequence CASP14 mode)	CASP14 targets	Lower performance compared with AlphaFold2 and ESMFold	Moderate computational demand	Structural analysis and epitope mapping	Lower accuracy without MSA support	Hýsková et al. (2026
T-cell Epitope Prediction (HLA Class I)	NetMHCpan 4.1	Pan-allele MHC-I binding prediction	Avg. Precision 0.868	24 HLA-I alleles; eluted ligand benchmark	Strong benchmark performance but below deep-learning models	Pan-allele imputation framework	CD8+ T-cell vaccine design	Reduced performance on rare HLA alleles	Wohlwend et al. (2025)
T-cell Epitope Prediction (HLA Class I)	MHCflurry 2.0	MHC-I binding affinity prediction	Avg. Precision 0.867; AUC 0.911 (9-mer benchmark)	24 HLA-I alleles; synthetic and naturally processed epitopes	ANN-based model outperformed regression-based tools	Open-source; 8.6× faster than NetMHC	Multi-allele vaccine design	Bias toward canonical anchor residues	Wohlwend et al. (2025); Zhao and Sher (2018)
T-cell Epitope Prediction (HLA Class I)	MixMHCpred 2.2	MHC-I ligand prediction	Avg. Precision 0.854	24 HLA-I alleles; eluted ligand benchmark	Lower benchmark precision than MUNIS, NetMHCpan, and MHCflurry	Optimized for ligand presentation analysis	Epitope prioritization	Lower performance at high FPR thresholds	Wohlwend et al. (2025)
T-cell Epitope Prediction (HLA Class I)	MUNIS	HLA class I ligand presentation prediction using deep learning	Avg. Precision 0.894; ROC-AUC 0.980	24 HLA-I alleles; 650k+ peptide-HLA training pairs	Best overall performance among compared HLA-I predictors	Large-scale deep-learning framework	Novel epitope discovery and validation	Requires extensive high-quality training data	Wohlwend et al. (2025)
T-cell Epitope Prediction (HLA Class II)	BERTMHC	Transformer-based MHC-II binding prediction	ROC-AUC 0.882	MHC class II binding benchmark	Marginal improvement over NetMHCIIpan (0.877)	Attention-based interpretability	CD4+ T-cell epitope prediction	Limited performance gain over prior tools	Villanueva-Flores et al. (2025)
Low-Data Allele Prediction	MHCSeqNet2	MHC-I binding prediction for low-data alleles	AUC 0.9842	45 HLA alleles with fewer than 200 positive peptides	Substantially outperformed NetMHCpan and MHCflurry on rare alleles	Pre-training using SMSNet data	Rare HLA allele vaccine design	More complex training pipeline	Wongklaew et al. (2023)
Multi-tool MHC-I Benchmark (9-mer)	SMM, SMMPMBEC, ANN-based tools	MHC-I peptide binding prediction	AUC range 0.856–0.911	18 tools; 32 HLA-I and 24 HLA-II alleles	ANN-based models consistently outperformed regression-based methods	Classical machine-learning approaches	General epitope prediction benchmarking	Lower predictive performance than deep-learning approaches	Zhao and Sher (2018)

The key Comparative Findings from the table suggests that AlphaFold2 achieved the highest structural prediction accuracy (TM-score 0.96), although ESMFold provided substantially faster inference suitable for high-throughput antigen screening. MHCSeqNet2 showed a major advantage for low-data and underrepresented HLA alleles, highlighting the importance of equitable dataset representation in vaccine AI pipelines. Transformer-based methods such as BERTMHC improved interpretability and class II epitope prediction, although performance gains over established tools remained modest. ANN and deep-learning models consistently outperformed regression-based approaches in large-scale MHC benchmarking studies.

Deep learning models capable of predicting three-dimensional protein structures from sequence with near-experimental accuracy now provide detailed insight into epitope geometry, surface accessibility, and conformational dynamics. These predictions enable the rational stabilization of metastable antigens, such as viral fusion proteins, in their immunologically relevant conformations One of the other greater applications is the use of generative models to design protein sequences and structures that do not exist in nature, but they are stable and functionally active. These models allow the creation of synthetic immunogens that preserve essential immune-recognition features while eliminating liabilities such as aggregation, instability, or undesired immune responses. This approach is particularly valuable for pathogens with highly variable surface proteins, where conserved neutralizing epitopes can be embedded within engineered scaffolds that focus immune responses on protective targets.

### 5.2 AI-enhanced reverse vaccinology and antigen prioritization

Reverse vaccinology originally reframed antigen discovery by starting from pathogen genomes rather than cultured organisms (Rappuoli, 2000). AI serves as a powerful engine for this approach, capable of performing rapid, large-scale genomic analysis that screens entire proteomes to identify conserved proteins and predict immunogenic epitopes. The landmark success of this strategy was the development of the Bexsero vaccine against meningococcal B (Pizza et al., 2000; Serruto et al., 2012).

Modern RV platforms have evolved into sophisticated, AI-driven workflows that integrate multiple prediction tools into a single, streamlined pipeline (Hayawi et al., 2024). A leading example is the NERVE 2.0 (New Enhanced Reverse Vaccinology Environment) platform, which automates the analysis of bacterial proteomes to identify promising vaccine candidates (Conte et al., 2024). NERVE 2.0 leverages AI and machine learning to systematically filter and rank proteins based on a range of critical criteria, such as their predicted subcellular localization, adhesion properties, conservation across different pathogenic strains, and lack of homology to host proteins to minimize the risk of autoimmune reactions. By consolidating these complex analyses into an accessible, user-friendly web interface (Doytchinova and Flower, 2007), platforms like NERVE 2.0 make advanced computational vaccinology available to a broader range of researchers and exemplify the power of AI to accelerate the discovery phase of vaccine development (Vivona et al., 2006). 

Genomic, transcriptomic, proteomic, and metabolomic data are integrated using transformer-based architectures with embedding and attention mechanisms to learn complex biological representations. These models enable the predictive identification of vaccine candidates, interaction networks, and therapeutic molecules, highlighting the shift toward data-driven and generative vaccine design.

### 5.3 AI-assisted mRNA vaccine design

In addition to protein immunogen engineering, AI is increasingly important for the design of mRNA vaccine constructs, because the antigen must be optimized not only at the protein level but also at the nucleic-acid level. Once a protective antigen or epitope is selected, computational models can assist in designing an mRNA sequence that encodes the desired antigen while improving translational efficiency, molecular stability, manufacturability, and innate immune compatibility (Pardi et al., 2018). Unlike direct protein design, where AI primarily optimizes amino acid sequence, folding, epitope exposure, and structural stability, mRNA vaccine design requires optimization of codon usage, GC content, untranslated regions, RNA secondary structure, ribosome accessibility, nucleotide modifications, and degradation-prone motifs. Machine-learning and deep-learning models can therefore be used to predict mRNA half-life, translation efficiency, secondary structure, and immunostimulatory risk, allowing researchers to generate mRNA constructs that efficiently express the intended antigen in host cells (Y. Li et al., 2025). This distinction is particularly relevant for vaccine development because an antigen with strong predicted immunogenicity may still perform poorly if the mRNA molecule encoding it is unstable, inefficiently translated, or excessively activates innate RNA-sensing pathways. Thus, AI-enabled vaccine design now extends beyond identifying antigenic proteins to engineering the complete vaccine-encoding molecule, linking antigen discovery, sequence optimization, delivery compatibility, and immune performance within a unified computational workflow (Jin et al., 2025).

## 6 Systems vaccinology and immune response modelling

Protective immunity is a state of the immune system in which an individual is safeguarded against a specific disease or infection, typically following a primary exposure through vaccination or natural infection. It is developed by coordinated interactions across multiple immune compartments rather than from isolated molecular events. Classical vaccinology has traditionally approached this complexity through reductionist assays such as measuring antibody titers, cytokine levels, or T-cell frequencies in isolation. Unsupervised and semi-supervised learning approaches can identify molecular programs associated with successful vaccination, such as early innate activation pathways that foreshadow robust adaptive immunity. These signatures often generalize across vaccines and populations, suggesting conserved immunological principles that can guide rational vaccine design.

A key application is the stabilization of viral glycoproteins in their metastable pre-fusion conformation, which is the primary target for many broadly neutralizing antibodies. AI models can predict this unstable structure, allowing researchers to perform structure-based design, introducing specific mutations to “lock” the protein in this desired state (Baek et al., 2021; Jumper et al., 2021). This approach was famously applied to vaccines for Respiratory Syncytial Virus (RSV) and SARS-CoV-2 (Corbett et al., 2020; McLellan et al., 2013). Beyond static structural predictions, advanced AI frameworks integrated with molecular dynamics (MD) simulations enable the characterization of protein conformational dynamics over time (Baek et al., 2021; Jumper et al., 2021). This maps the flexibility and accessibility of conformational epitopes over time, allowing for a more realistic assessment of which epitopes are consistently exposed and most likely to be recognized by B-cell receptors (Corbett et al., 2020; Walls et al., 2020).

### 6.1 AI-driven multi-omics integration in systems vaccinology

**Figure 6:**
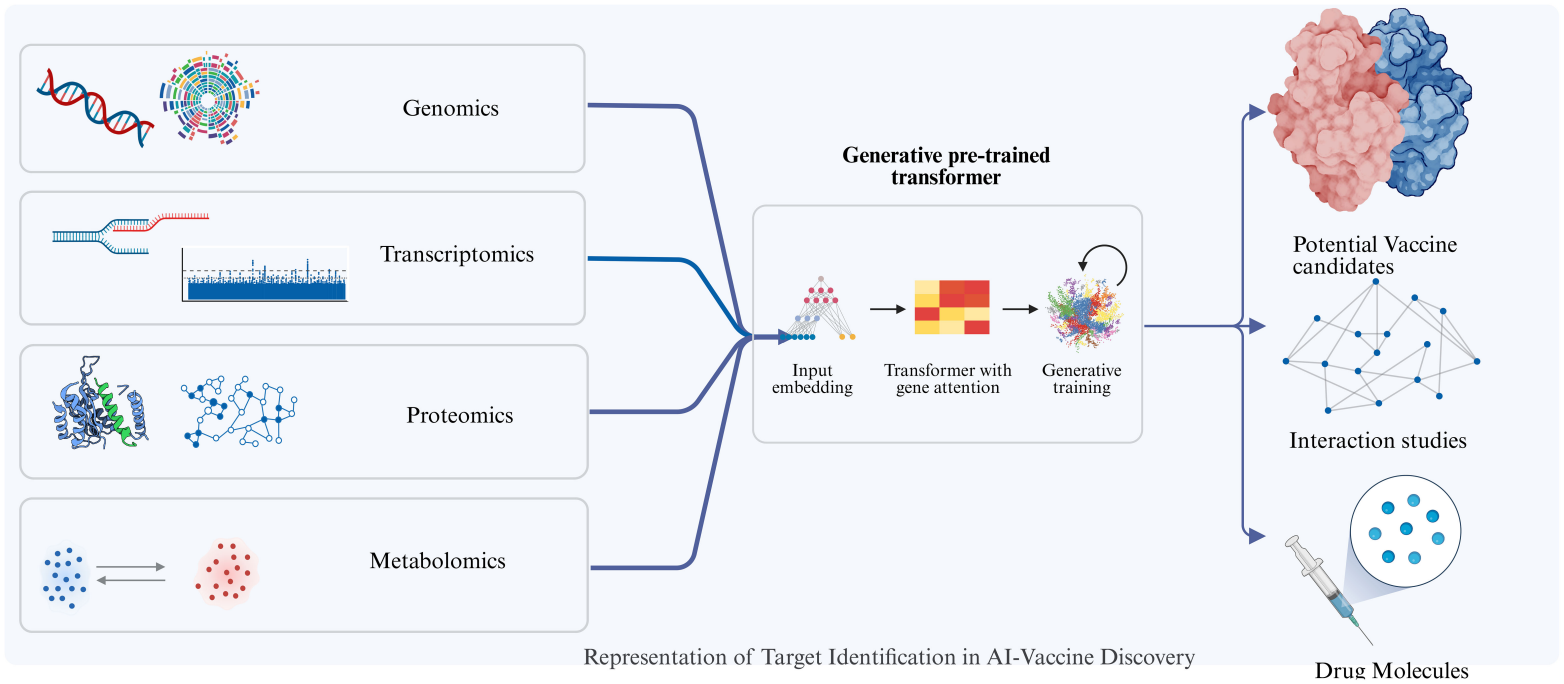
AI-driven multi-omics integration for vaccine target identification. The figure illustrates the integration of genomics, transcriptomics, proteomics, and metabolomics datasets into a generative pre-trained transformer (GPT)-based deep learning framework for identifying potential vaccine candidates, molecular interactions, and therapeutic drug molecules. The workflow demonstrates how multi-omics biological information is embedded and processed through transformer attention mechanisms and generative training models to facilitate predictive target discovery and computational therapeutic design. Created in BioRender. Nair, B. (2026) https://BioRender.com/43k62uo.

AI models are increasingly capable of moving beyond single-data-type analysis to integrate multi-omics datasets (genomics, proteomics, transcriptomics, metabolomics) as illustrated in (**Figure 6**) (Argelaguet et al., 2018; Natali et al., 2021). This system’s vaccinology approach provides a holistic view of pathogen-host interactions (Kösesoy et al., 2021; Natali et al., 2021). By identifying key nodes and patterns within these complex interaction networks, AI can pinpoint novel targets that are crucial for pathogen survival and visible to the immune system (Bravi, 2024; Waqas et al., 2023).

A range of computational methods are employed to uncover biologically relevant patterns within high-dimensional data. Classical machine learning models such as random forests and support vector machines serve this purpose alongside more complex deep learning architectures, including autoencoders and graph neural networks (GNNs) (Asediya et al., 2024; Hederman and Ackerman, 2023). Platforms like MOFA+ (Multi-Omics Factor Analysis) exemplify tools capable of integrating these diverse data layers, shifting vaccine discovery from a single-target approach toward a comprehensive, systems-level framework (Argelaguet et al., 2018).

### 6.2 AI-based modeling of T-cell dynamics and immune memory formation

Protective immunity extends beyond antibodies to include cytotoxic and helper T-cell responses, especially for intracellular pathogens and cancer vaccines (Kaech and Cui, 2012; Peters et al., 2020). The formation of long-lived memory T cells depends on complex interactions between antigen exposure, metabolic programming, and surrounding cytokine environment (Curtsinger and Mescher, 2010). AI models trained on longitudinal immune datasets distinguish effector versus memory T-cell fate by capturing temporal dynamics in gene expression, cytokine signaling, and metabolic reprogramming during immune responses (Hagan et al., 2015). Through analysis of time-resolved transcriptomic and immunological profiles, these models identify signatures associated with short-lived effector differentiation versus durable memory formation.

At the population level, AI models can capture T-cell receptor (TCR) repertoire diversity, providing insights into how vaccination reshapes clonal expansion and contributes to cross-reactive immunity against related pathogens (Emerson et al., 2017) These repertoire-level dynamics are directly linked to the formation and persistence of memory T cells, as clonal selection and expansion determine long-term immune protection. An emerging extension of this approach is the concept of immune digital twins which are computational models of individual or population-level immune systems that simulate T-cell responses and memory formation under different vaccination scenarios (Viceconti et al., 2016). By integrating genetic background, immune history, and environmental context, these models aim to predict personalized vaccine-induced immune memory in silico (Pollard and Bijker, 2020). Although still emerging, immune digital twins reflect the shift toward predictive modeling of T-cell responses and immune memory (Viceconti et al., 2016), with potential to enable more precise and personalized vaccine design.

## 7 AI-driven clinical trial optimization and real-world deployment

Clinical development remains the most complex and failure-prone stage of the vaccine pipeline. Despite promising preclinical findings, many candidates fail during human trials due to limited efficacy, safety concerns, or suboptimal study design (Wouters et al., 2021). Artificial intelligence is increasingly being applied in this phase to support adaptive trial design, optimize participant stratification, and enhance real-time data analysis (Abdolkhani et al., 2022; Bertsimas and Kallus, 2020). Rather than merely accelerating recruitment or statistical evaluation, computational approaches contribute to improved evidence generation through predictive modelling, anticipatory safety monitoring, and identification of early correlates of protection (Tsang et al., 2014). These developments aim to reduce uncertainty during clinical development while preserving scientific rigor and regulatory standards (Topol, 2019).

### 7.1 Clinical trials and participant stratification

A persistent bottleneck in vaccine trials is the identification and enrollment of appropriate participant cohorts (Wouters et al., 2021). AI algorithms can integrate epidemiological trends, electronic health records, demographic data, and mobility patterns to predict where and when infection risk will be highest, thereby enabling strategic site selection and targeted recruitment (Abdolkhani et al., 2022; Bertsimas and Kallus, 2020). This capability increases the likelihood that efficacy endpoints are reached efficiently. Beyond coordination, computational models support participant stratification based on baseline immune status, age, comorbidities, and genetic background (Tsang et al., 2014). By identifying subpopulations likely to respond differently to vaccination, trials can be designed to detect efficacy signals with smaller cohorts while preserving statistical power (Pollard and Bijker, 2020). Such stratification is particularly valuable for vaccines targeting heterogeneous populations or pathogens with uneven geographic distribution.

### 7.2 Adaptive and predictive clinical trials

Adaptive and Predictive Clinical Trials is where AI supports the implementation of adaptive clinical trial designs, in which parameters such as dosing, allocation ratios, or cohort composition are modified based on interim data analyses (Hutson, 2024). Machine learning models can evaluate emerging safety and immunogenicity signals in near real time, informing decisions to advance, modify, or discontinue trial arms (Bertsimas and Kallus, 2020). AI-driven participant stratification further enables identification of individuals who are likely to be high or low responders based on baseline immune, demographic, or clinical characteristics (Topol, 2019). Such approaches allow more targeted trial designs that may achieve statistical power with smaller, more precisely selected cohorts.

Predictive analytics further allow early inference of long-term outcomes from short-term immune readouts (Ankolekar et al., 2024). By learning relationships between early biomarkers such as antibody kinetics or transcriptional signatures and eventual protection (Haghayegh et al., 2024), AI can reduce reliance on prolonged follow-up, accelerating regulatory decision-making without compromising rigor (Nakaya et al., 2012). AI algorithms trained on data from historical vaccine trials can learn the correlation between initial immunological readouts (such as antibody titers at day 28) and the final clinical outcome (protection against infection a year later) (Khoury et al., 2021; Natali et al., 2021). It is thus possible to utilize these initial readouts as surrogate endpoints, with the prospect of accelerating evaluation and regulatory approval, as happened with the COVID-19 pandemic (Corbett et al., 2020).

### 7.3 Pharmacovigilance and real-time safety monitoring

The traditional practice of pharmacovigilance depends on passive surveillance systems like the Vaccine Adverse Event Reporting System (VAERS) that are prone to reporting delays, underreporting, and inconsistencies in data (Hazell and Shakir, 2006; Shimabukuro et al., 2015). AI-based systems revolutionize this paradigm fundamentally by allowing active, real-time safety monitoring (Fu et al., 2025; Huang et al., 2024). By correlating and analyzing heterogeneous streams of data ranging from Electronic Health Records (EHRs), clinical trials, insurance claims, to even social media computational methods can detect potential safety signals much earlier and with enhanced sensitivity (Ankolekar et al., 2024; Thakkar et al., 2023).

For example, natural language processing (NLP) can analyze clinician notes within EHRs to detect possible adverse events that are not reflected in structured diagnostic codes (Jensen et al., 2012; Wang et al., 2018). Statistical anomaly detection algorithms can monitor aggregated patient data continuously to alert statistical variation from projected event rates (Bate and Edwards, 2006). During the COVID-19 vaccination campaign, such surveillance systems contributed to the rapid identification and investigation of rare but serious adverse events, including vaccine-induced immune thrombotic thrombocytopenia (VITT) associated with adenoviral vector vaccines and myocarditis/pericarditis following mRNA vaccination (Greinacher et al., 2021; Oster et al., 2022). These near–real-time monitoring frameworks provide timely feedback to regulatory authorities and trial sponsors, supporting evidence-based protocol modifications and reinforcing participant safety and public confidence (Sharma et al., 2022; Topol, 2019).

### 7.4 Regulatory and ethical considerations

Critically analysing the AI-driven recruitment, stratification, and adaptive trial designs promise efficiency and precision, yet they introduce risks of bias, inequity, and regulatory friction as summarised in (**Table 3**). Predictive analytics and surrogate endpoints can accelerate approval, but overreliance on algorithmic correlations may compromise generalizability and long-term safety. Similarly, AI-enhanced pharmacovigilance offers real-time monitoring, but the integration of heterogeneous data streams raises challenges of signal reliability, privacy, and global harmonization. Overall, while AI can reduce uncertainty and improve evidence generation, its success depends on transparent validation, ethical governance, and careful alignment with regulatory standards to ensure that acceleration does not come at the expense of scientific rigor or public trust. 

**Table 3:** Bias analysis and validation framework of AI models in vaccine development.

Model/tool	Bias type/validation type	Evidence/validation details	Performance or key finding	Implication for vaccine development	Remaining gap/limitation	Source
AlphaFold2	Pathogen antigenic drift bias; External/prospective validation	Benchmarked on 1,337 PDB structures deposited after training cutoff; trained primarily on static PDB structures	High structural prediction accuracy across diverse proteins	Useful for structural vaccinology and antigen modelling	Limited ability to model rapidly mutating RNA viruses; reduced accuracy for IDPs and NMR structures	(Hýsková et al., 2026)
RoseTTAFold	Pathogen antigenic drift bias	Static structure-based training without host-pathogen co-evolution modelling	Lower structural prediction accuracy compared with AlphaFold2	Applicable for protein structure analysis	Poor generalization for rapidly evolving viral antigens	(Hýsková et al., 2026)
NetMHCpan 4.1	Training-data under-representation; Cross-validation and external benchmark validation	5-fold cross-validation on IEDB datasets; external benchmarking on 24 HLA-I alleles	Strong performance on common alleles	Supports CD8+ T-cell vaccine design	Reduced performance for rare HLA alleles and underrepresented populations	(Wohlwend et al., 2025; Wongklaew et al., 2023)
MHCflurry 2.0	Training-data bias and label heterogeneity; Cross-validation and external validation	Evaluated on synthetic and naturally processed peptide benchmarks	AUC 0.911 on 9-mer benchmark; faster than NetMHC	Efficient multi-allele vaccine prediction	Sensitive to allele-specific data imbalance and assay variability	(Wohlwend et al., 2025; W. Zhao and Sher, 2018)
MUNIS	Algorithmic opacity (black-box DL); External and prospective experimental validation	EBV CD8+ T-cell epitopes were validated *in vitro*.	ROC-AUC 0.980; experimentally validated novel epitopes	Strongest evidence base among epitope prediction models	Requires extensive training data and lacks interpretability	(Wohlwend et al., 2025)
BERTMHC	Algorithmic opacity; Cross-validation	Attention weights compared with known anchor residues	ROC-AUC 0.882 for MHC-II prediction	Improved interpretability for CD4+ T-cell epitope prediction	No prospective experimental validation	(Villanueva-Flores et al., 2025)
MHCSeqNet2	Training-data imbalance for rare alleles	Evaluated on 45 low-data HLA alleles (<200 positives)	AUC 0.9842 on rare allele datasets	Improves prediction equity for underrepresented HLA groups	More complex pre-training pipeline	(Wongklaew et al., 2023)

### 7.5 AI-driven vaccine manufacturing and process optimization

Vaccine manufacturing is an intrinsically complex biological process in which minor deviations in culture conditions, formulation, or handling can profoundly affect yield, stability, and quality. Traditionally, process optimization has relied on iterative experimentation and retrospective quality control. AI is transforming this paradigm by enabling predictive, real-time control of biomanufacturing systems (Plotkin et al., 2017).

Vaccine manufacturing involves a series of tightly controlled biological steps where even minor changes can affect yield and quality (Rosa et al., 2021). A bioreactor is at the heart of this process, where it provides a controlled environment for the growth of cells or microorganisms that produce the key vaccine components, such as viral particles, proteins, or mRNA templates (Fang et al., 2022). Parameters like temperature, pH, oxygen level, and nutrient supply must be maintained precisely, as they directly influence cell growth and the amount of antigen produced (M. Li and Qiu, 2013). Modern vaccine production uses large-scale bioreactors equipped with sensors and monitoring systems that collect data on each step of the process (Killeen et al., 2023). This information helps researchers understand how different conditions affect vaccine quality. By studying these patterns, scientists can adjust factors such as nutrient feeding time or mixing speed to improve consistency and efficiency (Yang et al., 2023).

For example, optimizing nutrient supply during the cell-culture phase can lead to higher antigen expression while maintaining stability and purity (Seo and Song, 2025).These controlled and data-informed manufacturing processes reduce waste, shorten development time, and ensure that each vaccine batch meets the required safety and quality standards all crucial for large-scale and reliable vaccine supply (Thakkar et al., 2023). Modern vaccine production facilities generate continuous streams of data from bioreactors, sensors, and quality assays (Suksaeree, 2025). Machine learning models can integrate these data to predict how variations in parameters such as temperature, pH, oxygen tension, and nutrient supply influence product consistency. By identifying non-linear interactions that elude conventional statistical methods, AI supports proactive intervention preventing batch failure rather than responding after the fact (Helleckes et al., 2023). This shift toward intelligent process control reduces waste, accelerates scale-up, and enhances reproducibility, all of which are critical for rapid response during public health emergencies (Suksaeree, 2025). The integration of AI into vaccine development demands parallel evolution in regulatory science. While regulatory agencies increasingly recognize the potential of AI-assisted approaches, acceptance hinges on demonstrable reliability, reproducibility, and transparency. Validation frameworks must ensure that AI models perform consistently across populations, pathogens, and contexts. Independent benchmarking, external validation datasets, and stress-testing against emerging variants are essential to mitigate overfitting and ensure generalizability (Alelyani, 2025). Regulators also require clear documentation of model architecture, training data, and performance metrics to support auditability (Mirakhori and Niazi, 2025). Explainability remains a central concern, particularly for deep learning systems. Efforts to develop interpretable AI models and standardized reporting guidelines will be critical for integrating AI outputs into regulatory decision-making without undermining confidence in vaccine safety (Alkhanbouli et al., 2025).AI introduces new ethical considerations into vaccinology, particularly regarding fairness, transparency, and accountability (Hanna et al., 2025). Models trained on datasets skewed toward high-income or majority populations risk perpetuating inequities in vaccine performance and access. Proactive bias detection and mitigation are therefore essential components of responsible AI deployment (Mehrabi et al., 2022). Ethical governance frameworks must also address data privacy and consent, especially as AI increasingly relies on sensitive genomic and clinical information. Compliance with international data protection standards is necessary but insufficient; meaningful public engagement and transparency are equally important for maintaining trust (Floridi et al., 2018). Importantly, ethical oversight should not be treated as an external constraint but as an integral design principle guiding AI-enabled vaccine development from inception through deployment (Olawade et al., 2024). Perhaps the most consequential question facing AI-driven vaccinology is whether it will exacerbate or reduce global health disparities. While AI has the potential to accelerate vaccine development and optimize distribution, these benefits will not be realized equitably without deliberate intervention (Niazi, 2025). Capacity-building initiatives that support data generation, computational infrastructure, and workforce training in low- and middle-income countries are essential (Okolo, 2022). Open-source platforms, federated learning approaches that preserve data sovereignty, and international collaboration can help democratize access to AI-enabled tools. Equitable vaccinology also requires aligning technological innovation with local health priorities, epidemiological realities, and community engagement (Akuma et al., 2025). AI-driven systems must therefore be embedded within broader global health strategies rather than deployed as isolated technological solutions (Akuma et al., 2025; Okolo, 2022).

## 8 Discussion

AI-driven methods have greatly sped up several stages of vaccine development, especially antigen discovery, epitope prediction, and immunogen design. Structure-prediction models like AlphaFold and RoseTTAFold allow for precise modeling of protein shapes (Jumper et al., 2021). This helps in identifying conformational B-cell epitopes and designing structurally stable antigens. Immunoinformatic tools, such as NetMHCpan and the IEDB Analysis Resource, enable quick large-scale screening of peptide candidates based on their predicted binding strengths to various HLA alleles. These tools proved especially useful during the COVID-19 pandemic, where AI-assisted reverse vaccinology pipelines sped up the discovery of immunogenic regions in SARS-CoV-2 proteins.

Recent advancements in generative and sequence-based AI models have broadened the field of computational vaccinology. Transformer-based models, like ProtBERT and ESM-2 (Lin et al., 2023), trained on large protein sequence sets, allow for predictions regarding protein function, the effects of mutations, and the design of new antigens. These models can explore sequence variations beyond what occurs in nature. AI has also played a key role in improving mRNA vaccine platforms (Brandes et al., 2022). Companies like Moderna and BioNTech have used machine learning to optimize codon usage, RNA secondary structure, and the stability of transcripts. Platforms like DeepVacPred show the benefits of merging machine learning with immunoinformatics to create multi-epitope vaccine designs with better immune responses.

Despite these advancements, several significant challenges affect the dependability and practical use of AI in vaccine development. A major issue is the lack of clarity in complex models, especially deep neural networks and transformer-based systems such as ProtBERT and ESM-2. These “black-box” models limit our understanding of their workings, making biological validation harder and lowering trust in their predictions. Additionally, biases in training data can carry over into predictive models like NetMHCpan and DeepVacPred, potentially causing inaccuracies for specific populations, especially for rare HLA alleles (Reynisson et al., 2020).

Another significant challenge is the need for high-quality, tested datasets. Resources such as the IEDB Analysis Resource depend heavily on curated epitope data, which can be limited or non-existent for newer or less understood pathogens. Faulty or incomplete datasets can result in false-positive and false-negative predictions, undermining vaccine design. Also, many AI models depend on fixed datasets and may not adjust well to quickly changing pathogens, especially RNA viruses that undergo antigenic drift and shift (Dhanda et al., 2019).

Ethical and regulatory issues make it harder to integrate AI into vaccine development. Using sensitive human genomic and clinical data raises privacy and security concerns. Quick timelines from AI-driven methods may exceed the pace of current regulatory systems, which are not fully prepared to assess AI-assisted design pipelines. Relying too much on computational predictions without enough experimental validation raises the risk of failure in later clinical stages.

Additionally, current AI models often struggle with generalization. While AlphaFold and RoseTTAFold excel at predicting static protein structures, these models do not consider dynamic immune interactions, antigen processing pathways, post-translational modifications, or the co-evolution of hosts and pathogens (Jumper et al., 2021). Therefore, predictions should be linked to experimental immunology, highlighting the need for combined frameworks that integrate AI predictions with ongoing lab validation and optimization.

Future improvements should target better understanding through explainable AI methods, allowing for clearer insights into model predictions. Developing diverse, high-quality, and continuously updated datasets is crucial for reducing bias and enhancing reliability. Incorporating multi-omics data, like genomics, proteomics, and immunopeptidomics, will further improve prediction accuracy. Establishing standardized regulations for AI-assisted vaccine design is vital to ensure reproducibility, safety, and ethical practices.

In summary, AI offers both benefits and risks in vaccine development. Tools like AlphaFold, RoseTTAFold, NetMHCpan, ProtBERT, ESM-2, and DeepVacPred have significantly changed how vaccines are developed. However, effective implementation demands thorough validation, quality data, and strong regulatory oversight. A balanced approach that combines computational predictions with experimental validation is crucial for the responsible progress of next-generation vaccines.

## 9 Conclusion

Artificial intelligence has emerged as a transformative force in vaccine development, offering unprecedented capabilities to accelerate discovery, optimize design, enhance safety and improve global distribution. The successful integration of AI across the vaccine development pipeline from initial antigen discovery through manufacturing and public health communication demonstrates the technology’s potential to revolutionize immunological research and pandemic preparedness.

The comprehensive implementation of AI in vaccine development requires coordinated efforts across multiple domains: robust data infrastructure, advanced computational resources, integrated workflows, and adaptive regulatory frameworks. While significant challenges remain including data quality issues, model interpretability concerns, and equity considerations the continued evolution of AI technologies and their successful application during the COVID-19 pandemic provides a solid foundation for future advances.

Looking forward, the integration of AI with emerging technologies, the development of personalized vaccination strategies, and the establishment of global AI-driven health systems promise to further enhance vaccine development capabilities. Success in realizing these benefits will require sustained investment in infrastructure, stakeholder engagement, transparent model documentation, and continuous attention to ethical considerations and global health equity.

As the field continues to evolve, AI-driven vaccine development represents not merely a technological advancement, but a fundamental paradigm shifts toward more precise, efficient, and equitable approaches to global health protection. The lessons learned from recent successes and ongoing challenges will inform the development of next-generation AI systems capable of addressing emerging health threats with unprecedented speed and effectiveness.
